# Drug-Induced Bullous Pemphigoid Triggered by Immune Checkpoint Inhibitor Opdualag in an Elderly Patient With Recurrent Metastatic Melanoma: A Case Report and Treatment Course With Dupilumab

**DOI:** 10.7759/cureus.97838

**Published:** 2025-11-26

**Authors:** Brian A Moreno, Moises Lutwak, Daniel Lutwak, Francisco Kerdel

**Affiliations:** 1 Dermatology, Lake Erie College of Osteopathic Medicine, Bradenton, USA; 2 Dermatology, Larkin Community Hospital, South Miami, USA; 3 Dermatology, Florida Academic Dermatology Center, Coral Gables, USA

**Keywords:** academic dermatology, bullous pemphigoid, clinical dermatology, complex medical dermatology, dermatology oncology, dermatology training, general dermatology, immune checkpoint inhibitors, medical dermatology, skin disease/dermatology

## Abstract

Bullous pemphigoid (BP) is an autoimmune subepidermal blistering disorder most commonly affecting elderly individuals and may be triggered by medications, including immune checkpoint inhibitors (ICIs). We present the case of an 81-year-old male with a history of recurrent metastatic melanoma previously treated with nivolumab and relatlimab (Opdualag), who developed a persistent pruritic eruption following initiation of dual PD-1/LAG-3 blockade. Despite discontinuation of immunotherapy six months before dermatologic evaluation and use of multiple topical corticosteroids and oral antihistamines, symptoms persisted. A punch biopsy and direct immunofluorescence demonstrated linear IgG and C3 deposition along the basement membrane zone, consistent with BP. On initial dermatologic examination, urticarial pink plaques involved approximately 50% of the body surface area, with lesions affecting the left medial inferior chest and epigastric region. Treatment with subcutaneous dupilumab was initiated alongside clobetasol 0.05% ointment. At follow-up, the patient reported persistent activity, receiving intramuscular triamcinolone acetonide and adjunctive therapies, including hydroxyzine, hydrocortisone 2.5% cream (for groin), and triamcinolone 0.1% ointment. By the second week of treatment, new plaques continued to appear daily without subjective improvement. Objective scoring with the Bullous Pemphigoid Disease Area Index was not obtained. This case illustrates the diagnostic and therapeutic challenges in managing ICI-induced BP and highlights the early course of dupilumab therapy, which had not yet produced clinical improvement at the time of last follow-up. Continued therapy was planned, and no adverse effects were reported.

## Introduction

Bullous pemphigoid (BP) is the most common autoimmune subepidermal blistering disease in older adults, mediated by autoantibodies against hemidesmosomal proteins BP180 and BP230 [1]. Although traditionally idiopathic, BP can be triggered by medications, including immune checkpoint inhibitors (ICIs), which may induce or unmask autoreactivity to basement membrane zone antigens [1-4]. Recent reviews have noted increasing BP incidence in parallel with expanding ICI use in oncology [2].

ICIs, including PD-1 and PD-L1 inhibitors, have transformed the treatment landscape for multiple malignancies but are associated with a broad spectrum of immune-related adverse events (irAEs), including autoimmune blistering diseases [1-3]. Dual checkpoint blockade with nivolumab and relatlimab (anti-PD-1 + anti-LAG-3) has demonstrated significant antitumor efficacy in melanoma, as established in the RELATIVITY-047 trial and subsequent reviews [3], but may potentiate complex cutaneous toxicity.

ICI-related BP often presents with widespread pruritic plaques, may lack initial blister formation, and can persist despite discontinuation of immunotherapy due to prolonged immune activation [2,3]. Management is especially challenging in elderly patients or those with concurrent malignancy, where systemic corticosteroids or immunosuppressants may pose significant risks. Conventional steroid-sparing agents, including azathioprine, mycophenolate, and methotrexate, remain central options, as supported by recent real-world outcomes data [5].

Dupilumab, an IL-4Rα antagonist inhibiting IL-4 and IL-13 signaling, has shown promising efficacy in idiopathic and drug-induced BP with minimal systemic immunosuppression [6-15]. Multiple case series have reported improvement in pruritus, lesion count, and disease control, including in patients with ICI-associated BP [6-15].

This case represents one of the earliest documented instances of BP associated with PD-1/LAG-3 combination therapy and contributes to the growing evidence on dupilumab as a steroid-sparing option for ICI-induced BP.

## Case presentation

An 81-year-old male with a past medical history of malignant melanoma presented for a full-body skin examination. The histologic subtype of the melanoma was not available in the provided medical documentation. He was diagnosed with melanoma in 2014 and treated with pembrolizumab (Keytruda), achieving remission until 2024, when recurrence prompted initiation of dual immune checkpoint therapy with nivolumab and relatlimab (Opdualag). Approximately six months before presentation, he discontinued Opdualag due to a persistent pruritic eruption unresponsive to clobetasol, hydrocortisone, triamcinolone, and oral diphenhydramine.

**Figure 1:**
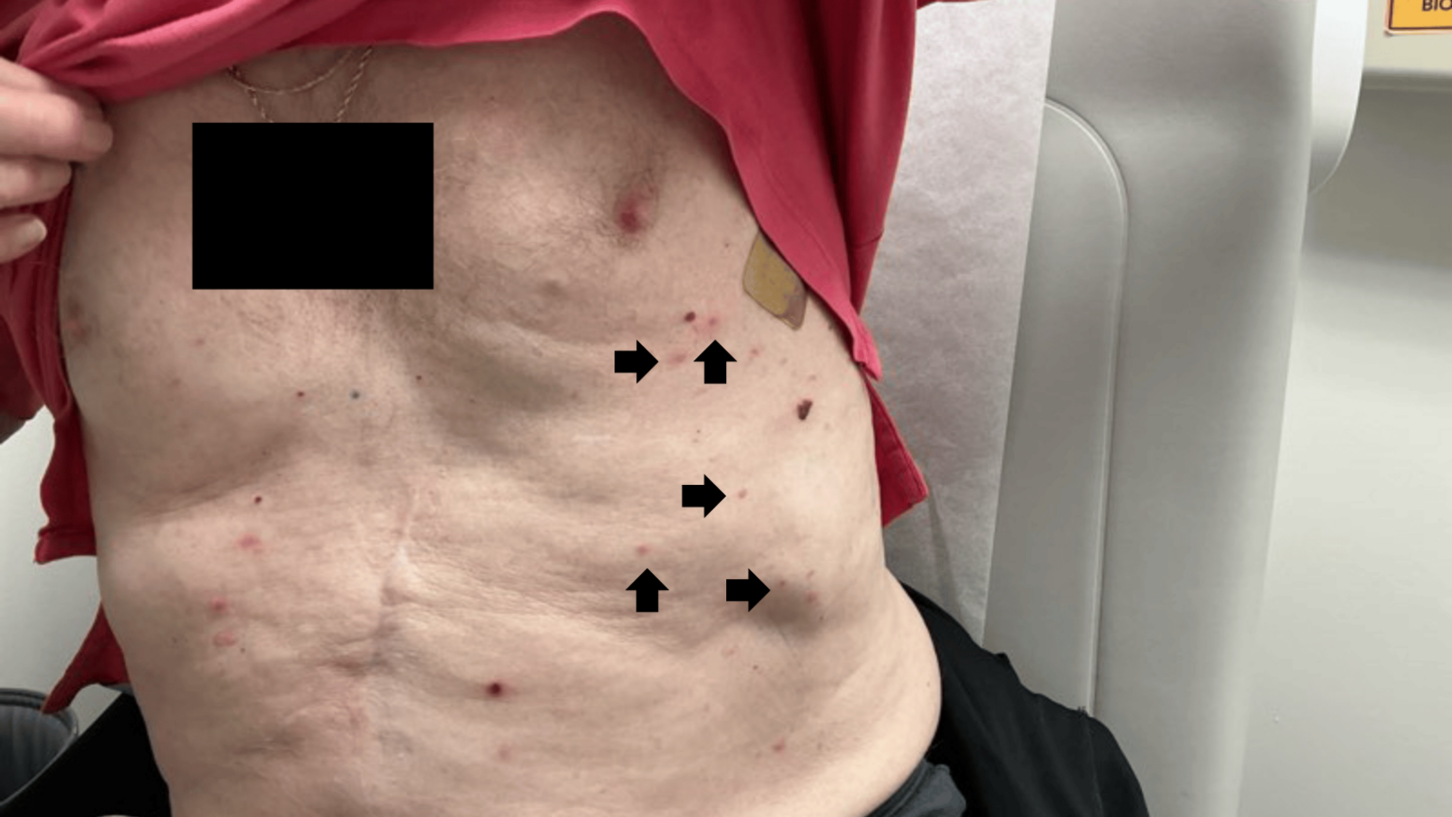
Urticarial pink plaques involving the left medial inferior chest and epigastric region (arrows indicate representative plaques). No bullae, vesicles, or erosions were present. This image corresponds to the initial dermatologic presentation and demonstrates the extent of trunk involvement.

On initial dermatologic evaluation, physical examination revealed widespread urticarial pink plaques involving the left medial inferior chest and epigastric region (Figure 1). The affected body surface area (BSA) was documented as 50%, consistent with broad anterior trunk involvement and scattered lesions on the extremities. Lesions consisted exclusively of urticarial plaques without tense bullae, vesicles, erosions, crusting, or mucosal involvement. Distribution later extended to the left clavicular neck and right arm. The patient reported ongoing development of one to two new plaques per day. Objective severity measures such as the Bullous Pemphigoid Disease Area Index (BPDAI) were not recorded. Pruritus was described as moderate, but no numerical pruritus score was documented.

**Figure 2:**
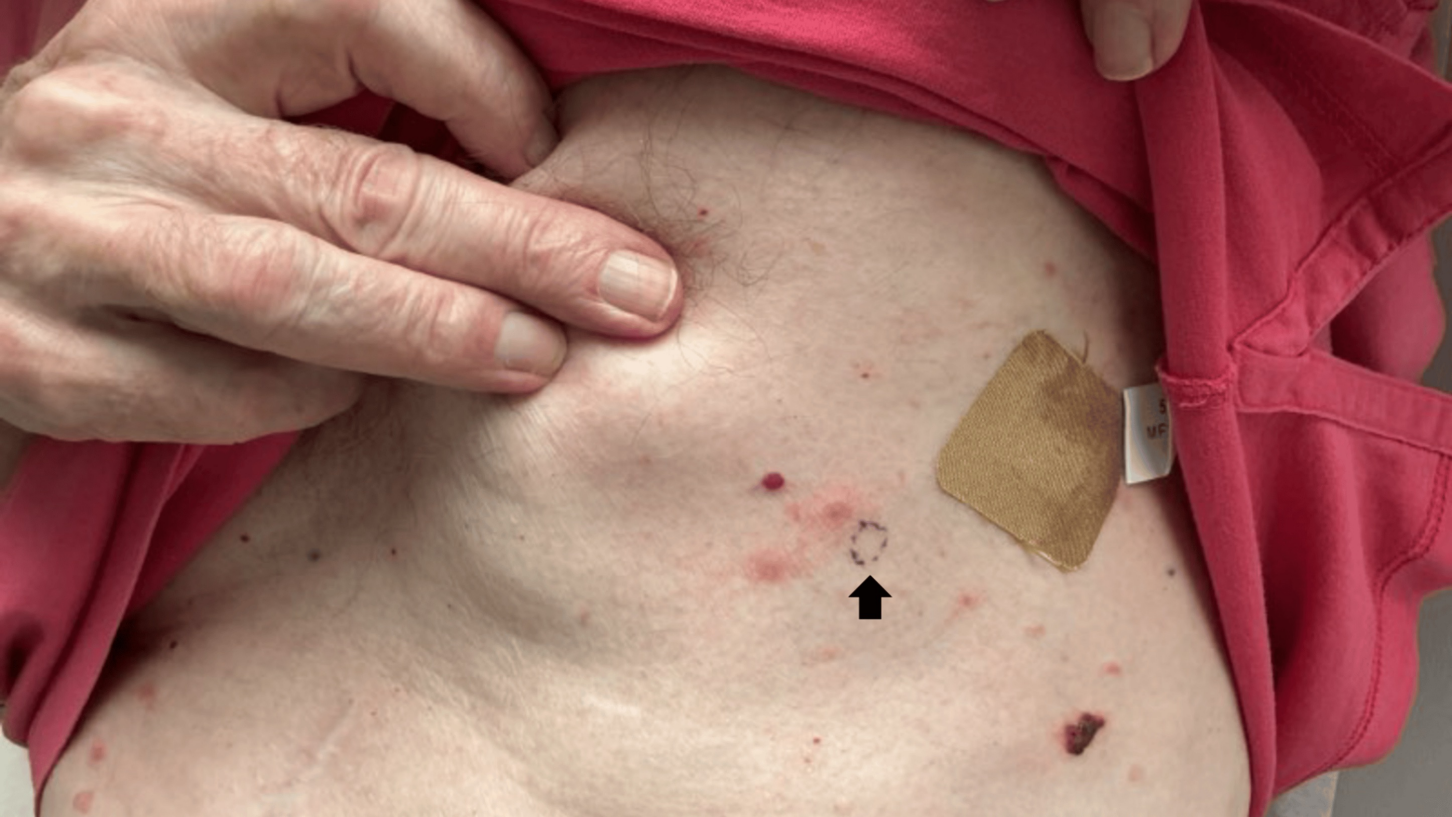
Perilesional punch biopsy site on the epigastric skin (arrow indicating biopsy location). This 6-mm biopsy was obtained for direct immunofluorescence evaluation, which revealed linear IgG and C3 deposition along the basement membrane zone consistent with bullous pemphigoid.

A perilesional 6-mm punch biopsy of the epigastric region was obtained for direct immunofluorescence (Figure 2), which demonstrated linear IgG and C3 deposition along the basement membrane zone, confirming BP. Staining intensity was not provided in the report. Outside pathology records corroborated the diagnosis, identifying a subepidermal blister with eosinophils from a biopsy of the left rib cage. BP180 and BP230 serologic studies were ordered; however, the results were not available. Given failure of topical therapies and continued symptomatic activity, dupilumab was initiated (600-mg loading dose followed by 300 mg every other week). Clobetasol 0.05% ointment and petrolatum wound care were prescribed for active lesions and the biopsy site, respectively.

At the one-week follow-up, the patient reported that dupilumab had not yet provided benefit. Urticarial plaques persisted on the left medial inferior chest, and he received intramuscular triamcinolone acetonide (40 mg). Hydroxyzine 25 mg was added for nocturnal pruritus, and hydrocortisone 2.5% cream was prescribed for groin involvement, where high-potency steroids were avoided due to thin skin.

**Figure 3:**
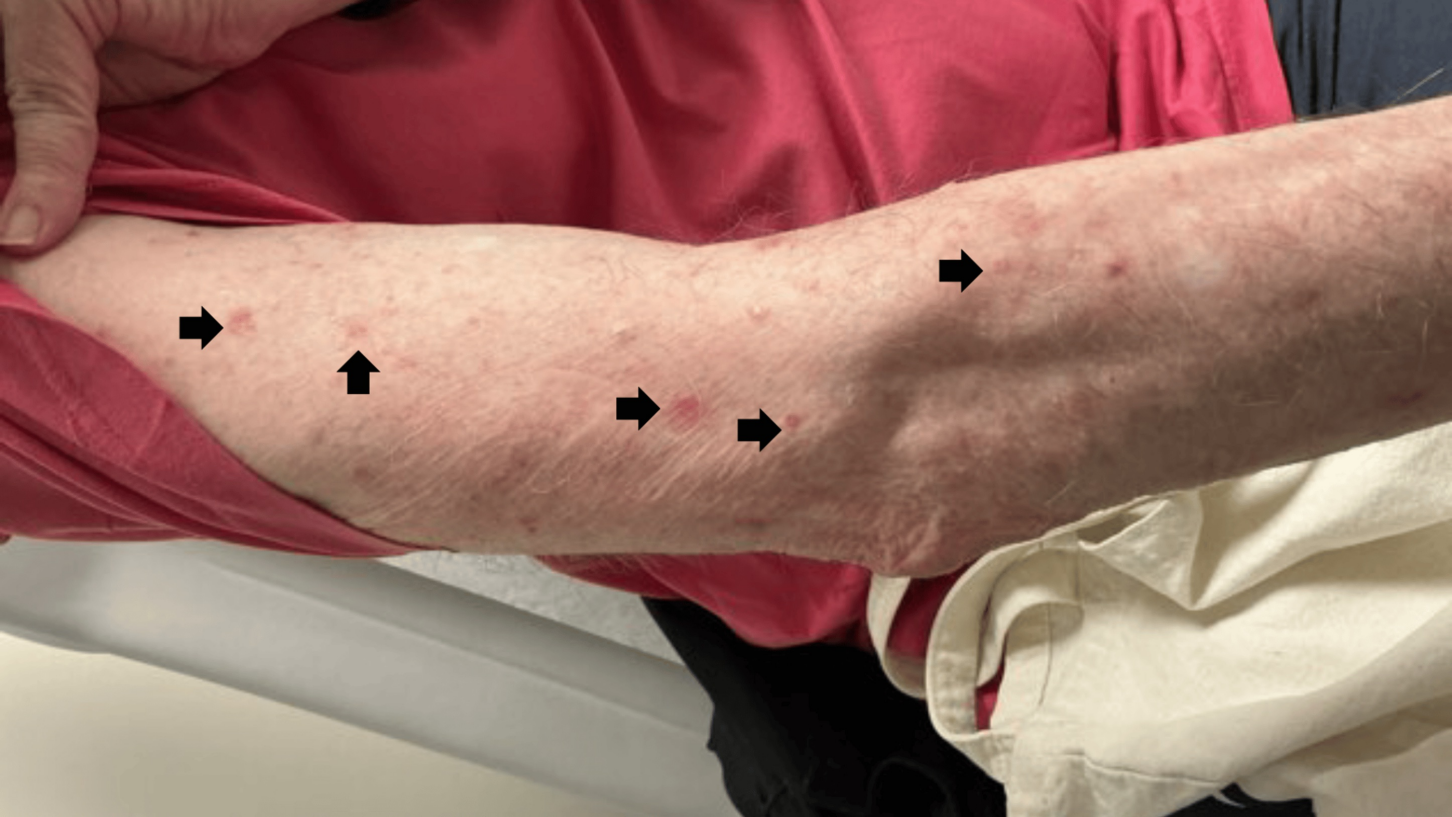
Persistent urticarial plaques on the right arm at the two-week follow-up visit (arrow indicating active lesion). Despite ongoing treatment with dupilumab and topical corticosteroids, the patient continued to develop one to two new plaques per day with no early improvement.

At the final available follow-up, approximately two weeks after initiating dupilumab, the patient continued to develop one to two new urticarial plaques daily with no subjective or objective improvement (Figure 3). Dupilumab therapy remained ongoing, and no adverse effects were reported. The patient continued applying clobetasol 0.05% to pruritic plaques, using CeraVe moisturizer after showering, and applying triamcinolone 0.1% ointment and a clobetasol-talcum powder mixture to excoriated (“open”) plaques. No additional follow-up documentation was available.

## Discussion

ICI-induced BP is believed to result from loss of immune tolerance and subsequent autoreactivity to BP180 and BP230, a mechanism supported by early clinical observations of PD-1/PD-L1-associated BP [1-4]. Dual PD-1/LAG-3 blockade has emerged as an effective melanoma therapy, but recent clinical experience and trial data indicate an associated rise in dermatologic toxicity [3].

This patient developed a non-bullous, urticarial variant of BP, a recognized early phenotype in ICI-related cases [1-3]. Management is often difficult in elderly oncology patients in whom systemic immunosuppression may jeopardize cancer therapy. Conventional steroid-sparing agents, including mycophenolate, azathioprine, and methotrexate, remain validated options for refractory disease, with real-world evidence describing their safety and outcomes in ICI-related cutaneous irAEs [5].

Dupilumab use has expanded following multiple reports demonstrating improvement in pruritus, disease activity, and the ability to continue cancer therapy in some patients with ICI-induced BP [6-15]. Although dupilumab is a mechanistically targeted and generally well-tolerated therapy, the present case includes only the initial two-week treatment interval, during which no improvement was observed. The absence of BPDAI scoring and limited follow-up restricts quantitative interpretation of therapeutic response. Continued monitoring and additional treatment cycles were planned, and therapy remained ongoing at the last documentation.

This case underscores the need for standardized severity metrics, including BPDAI, pruritus scales, and steroid-sparing quantification, to allow reproducible evaluation of emerging therapies such as dupilumab in ICI-associated BP. It also highlights the importance of recognizing early non-bullous presentations of BP in patients receiving checkpoint inhibitor therapy.

## Conclusions

ICI-induced BP is an increasingly recognized irAE, particularly with the expanding use of dual checkpoint blockade. This patient’s early, non-bullous presentation and persistent urticarial plaques highlight the diagnostic challenges associated with ICI-related BP. Dupilumab was initiated after failure of topical therapy, and although no early improvement was observed, treatment remained ongoing at the last follow-up. This case emphasizes the importance of prompt dermatologic evaluation, recognition of non-bullous BP patterns, and structured severity assessment when managing cutaneous irAEs. As reports accumulate, dupilumab may represent a valuable steroid-sparing option for patients with ICI-induced BP who are unable to tolerate systemic immunosuppression.

## References

[1] Asdourian MS, Shah N, Jacoby TV, Reynolds KL, Chen ST (2022). Association of bullous pemphigoid with immune checkpoint inhibitor therapy in patients with cancer: a systematic review. JAMA Dermatol.

[2] Shalata W, Weissmann S, Itzhaki Gabay S (2022). A retrospective, single-institution experience of bullous pemphigoid as an adverse effect of immune checkpoint inhibitors. Cancers (Basel).

[3] Albrecht LJ, Livingstone E, Zimmer L, Schadendorf D (2023). The latest option: nivolumab and relatlimab in advanced melanoma. Curr Oncol Rep.

[4] Naidoo J, Schindler K, Querfeld C (2016). Autoimmune bullous skin disorders with immune checkpoint inhibitors targeting PD-1 and PD-L1. Cancer Immunol Res.

[5] Ruf T, Kramer R, Forschner A (2024). Second-line therapies for steroid-refractory immune-related adverse events in patients treated with immune checkpoint inhibitors. Eur J Cancer.

[6] Guan S, Zhang L, Zhang J, Song W, Zhong D (2022). A case report of steroid-refractory bullous pemphigoid induced by immune checkpoint inhibitor therapy. Front Immunol.

[7] Kuo AM, Gu S, Stoll J (2023). Management of immune-related cutaneous adverse events with dupilumab. J Immunother Cancer.

[8] Nykaza I, Moy A, Dusza SW (2025). Dupilumab for bullous pemphigoid related to immune checkpoint inhibitors: a retrospective case series. Oncologist.

[9] Grüninger J, Lehr S, Meiss F, Rafei D, Schauer F (2025). Case report: dupilumab therapy for immune checkpoint inhibitor-induced bullous pemphigoid enables dual immunotherapy initiation in progressive malignant melanoma. Front Oncol.

[10] Zhang Y, Xu Q, Chen L (2021). Efficacy and safety of dupilumab in moderate-to-severe bullous pemphigoid. Front Immunol.

[11] Wang M, Wang J, Shi B (2022). Case report: dupilumab for the treatment of bullous pemphigoid. Dermatol Ther.

[12] Liang J, Abulikemu K, Maolidan Maolidan (2023). Nine cases of refractory bullous pemphigoid treated with dupilumab and literature review. Int Immunopharmacol.

[13] Planella-Fontanillas N, Bosch-Amate X, Jiménez Antón A (2025). Real-world evaluation of the effectiveness and safety of dupilumab in bullous pemphigoid: an ambispective multicentre case series. Br J Dermatol.

[14] Abdat R, Waldman RA, de Bedout V (2020). Dupilumab as a novel therapy for bullous pemphigoid: a multicenter case series. J Am Acad Dermatol.

[15] Jiang X, Chen L, Zhu Y, Zhan S, Jin N, Cheng H (2025). Effective response to high-frequency and prolonged dupilumab therapy in severe refractory bullous pemphigoid: a case study. Hum Vaccin Immunother.

